# Feeder-free differentiation of human iPSCs into natural killer cells with cytotoxic potential against malignant brain rhabdoid tumor cells

**DOI:** 10.1016/j.bioactmat.2024.02.031

**Published:** 2024-03-08

**Authors:** Sonia Kiran, Yu Xue, Drishty B. Sarker, Yan Li, Qing-Xiang Amy Sang

**Affiliations:** a Department of Chemistry and Biochemistry, Florida State University, Tallahassee, FL 32306-4390, USA; b Department of Chemical and Biomedical Engineering, FAMU-FSU College of Engineering, Florida State University, Tallahassee, FL, 32310-6046, USA; c Institute of Molecular Biophysics, Florida State University, Tallahassee, FL, 32306-4380, USA

**Keywords:** Human induced pluripotent stem cells, Natural killer cells, Atypical teratoid rhabdoid tumor, Cytotoxicity, Cytokine activation

## Abstract

Natural killer (NK) cells are cytotoxic immune cells that can eliminate target cells without prior stimulation. Human induced pluripotent stem cells (iPSCs) provide a robust source of NK cells for safe and effective cell-based immunotherapy against aggressive cancers. In this *in vitro* study, a feeder-free iPSC differentiation was performed to obtain iPSC-NK cells, and distinct maturational stages of iPSC-NK were characterized. Mature cells of CD56^bright^ CD16^bright^ phenotype showed upregulation of CD56, CD16, and NK cell activation markers NKG2D and NKp46 upon IL-15 exposure, while exposure to aggressive atypical teratoid/rhabdoid tumor (ATRT) cell lines enhanced NKG2D and NKp46 expression. Malignant cell exposure also increased CD107a degranulation markers and stimulated IFN-γ secretion in activated NK cells. CD56^bright^ CD16^bright^ iPSC-NK cells showed a ratio-dependent killing of ATRT cells, and the percentage lysis of CHLA-05-ATRT was higher than that of CHLA-02-ATRT. The iPSC-NK cells were also cytotoxic against other brain, kidney, and lung cancer cell lines. Further NK maturation yielded CD56^−ve^ CD16^bright^ cells, which lacked activation markers even after exposure to interleukins or ATRT cells - indicating diminished cytotoxicity. Generation and characterization of different NK phenotypes from iPSCs, coupled with their promising anti-tumor activity against ATRT *in vitro*, offer valuable insights into potential immunotherapeutic strategies for brain tumors.

## Highlights

• Natural killer (NK) cells were derived from human induced pluripotent stem cells (iPSCs) in the absence of feeder cells.
• Various maturational subtypes of iPSC-NK cells were characterized, and the phenotypic and functional properties were studied.
• iPSC-NK cells of CD56^bright^ CD16^bright^ phenotype expressed activation markers in response to interleukin stimuli.
• iPSC-NK cells were cytotoxic toward human atypical teratoid and rhabdoid tumor (ATRT) cells and other human cancer cells.
• The cytotoxicity of iPSC-NK cells against various cancer cells *in vitro* might be translated into an *in vivo* immunotherapy.

## Graphical abstract

**Figure:**
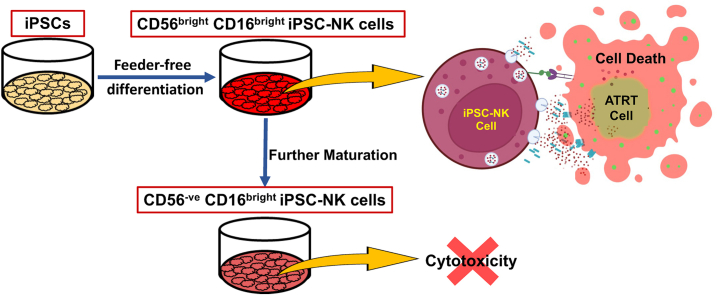

## 1 Introduction

Natural killer (NK) cells are innate immune cells that recognize and target non-self-cells, such as cancer and virus-infected cells, without prior sensitization [[1], [2], [3]]. They use their activation and inhibition receptors to sense the non-self cells and employ cell death pathways, such as the perforin-granzyme B complex and FasL-TRAIL activation pathway, to attack them [4,5]. Human NK cells have multiple subtypes based on maturity and cytolytic activity - an understanding of what is particularly important for deriving functionally mature NK cells from stem cell sources. The CD56^bright^ CD16^−ve^ subset of NK cells is found mainly in secondary lymphoid tissue and comprises cytokine-producing immature NK cells [[6], [7], [8]]. CD56^−ve^ CD16^bright^ and CD56^bright^ CD16^bright^ are cytolytic NK cells and populate human peripheral blood [[7], [8], [9]]. The CD56^−ve^ CD16^bright^ phenotype belongs to a minor subtype of NK cells that displayed impaired cytolytic function with aging [10]. Experimental evidence suggests that CD56^bright^ NK cells give rise to CD56^−ve^ cells during maturation [2,7].

Cell-based anticancer immunotherapies, such as T cell- and NK cell-based therapies, are of increasing interest for their promising therapeutic potential. Although T cells are highly effective in killing their targets, the requirements of T cell priming and human leukocyte antigen (HLA) restriction limit their+ usefulness [11,12]. The function of innate immune cells, such as NK cells, is not impacted by HLA restriction. They have shown promising results because of their lower risk of graft versus host disease (GVHD) and cytokine release syndrome (CRS) and higher graft versus tumor potential [13]. However, the main concern regarding allogenic and autologous NK cell immunotherapy is the limited number of available cells, resulting in poor access to adoptive cell therapy. This limitation can be addressed by human pluripotent stem cell-derived NK cells, which can provide a robust source of off-the-shelf NK cells and can be more accessible for cancer treatment [14].

Some common approaches to derive NK cells by differentiating human pluripotent stem cells use murine stromal cells as feeders [[14], [15], [16], [17], [18], [19]]. However, the derived NK cells face hurdles in their clinical application because of the presence of murine cells and associated contaminants [[20], [21], [22]]. Therefore, the implementation of a feeder-free method to differentiate human induced pluripotent stem cells (iPSCs) into NK cells would be highly desirable. Reports showed that iPSC-derived NK cells have high proliferative potential and desired homogeneity for therapeutic use [23]. Furthermore, since iPSCs are amenable to genome editing, modified NK cells with high target specificity, persistence, and immune activation potential are achievable [[24], [25], [26]]. Three clinical trials are ongoing to evaluate the safety and efficacy of iPSC-NK cells in adult patients with various solid and hematologic malignancies (NCT03841110, NCT04023071, and NCT04630769) [24]. Despite ongoing therapeutic and clinical development, the use of NK cells in the treatment of pediatric cancers, especially rare pediatric brain tumors, is largely underexplored [27].

Atypical teratoid rhabdoid tumor (ATRT) is an aggressive human pediatric tumor of the central nervous system (CNS). Also known as malignant rhabdoid tumors of the brain, ATRT accounts for 20% of all CNS tumors in children under age 3 [22,28,29]. This pediatric brain tumor occurs due to the biallelic inactivating mutations of the SMARCB1 (SWI/SNF related, Matrix Associated, Actin Dependent Regulator of Chromatin, subfamily B, member 1) gene in chromosome 22 [30]. The current ATRT treatment approach includes surgery followed by chemotherapy [31] and radiotherapy withstem cell rescue [32]. Although almost 50% of patients receiving multimodal therapy survive at least four years [33], adjuvant chemotherapy and radiotherapy can lower long-term neurocognitive performance [34] and cause reproductive and systemic dysfunction in young ATRT patients [35,36]. Therefore, there is currently an urgent need to develop biologically targeted therapies that can replace existing treatment regimens to improve the long-term survival of these pediatric patients.

Toward that goal, a variety of immunotherapies are in development to treat ATRT. These immunotherapies include dendritic cell-based vaccines and T cell-based immunotherapies, which are undergoing investigation in pre-clinical and clinical trials [[37], [38], [39]]. However, the complexity of production and inter-individual variation are some intrinsic limitations of these modalities [40,41]. iPSC-derived NK cell therapy overcomes these constraints for safer clinical use [42] and has the potential to advance further [27,43]. Moreover, innate NK cells have been shown to infiltrate the immune microenvironment of the majority of ATRTs, placing greater importance on their antitumor potential against brain malignancies [39]. Therefore, in this study, we aimed to obtain different maturational subtypes of iPSC-NK cells and test their *in vitro* efficacy against malignant cells, which may benefit the future exploration of NK cell therapy.

The hypothesis is that NK cells derived from human iPSCs through feeder-free differentiation are cytotoxic toward malignant brain rhabdoid tumor cells, and the effects can be increased via prior activation with interleukins. Here, iPSC-NK cells were allowed to mature and develop a cytotoxic phenotype. Mature iPSC-NK cells were then cocultured with ATRT cells at various effector-to-target ratios to test their functional cytotoxicity. This iPSC-NK cell differentiation strategy to obtain functionally mature cytotoxic cells, along with their antitumor activity against ATRT cells *in vitro*, offers valuable insights into potential immunotherapeutic strategies for brain tumors.

## 2 Materials and methods

### 2.1 Human iPSC and cancer cell culture

The Episomal (Epi)-iPSCs (Thermo Fisher, Cat# A18945) were used for NK differentiation. These cells are derived from CD34^+^ cord blood using a three-plasmid episomal system that codes for seven reprogramming factors (SOX2, OCT4, KLF4, MYC, NANOG, LIN28, and SV40 T antigen; SOKMNLT) [44]. The cells were plated with a surface density of 1 × 10^6^ cells per well on a 6-well plate coated with Matrigel matrix (Life Technologies). They were maintained in mTeSR plus serum-free medium (Stem Cell Technologies, Inc). For the initial 24 h, growth maintaining factor called Rho-associated protein kinase (ROCK) inhibitor Y27632 (10 μM, Sigma) was added.

In this study, two distinct types of ATRT cell lines, namely CHLA-05-ATRT (ATCC® CRL-3037™, ATCC) and CHLA-02-ATRT (ATCC® CRL-3020™, ATCC), were used to test the cytotoxic effect of iPSC-NK cells. The ATRT cells were cultured in suspension on a non-treated surface using Gibco Dulbecco's Modified Eagle Medium: Nutrient Mixture F-12 (DMEM/F12, Thermo Fisher Scientific, Waltham, MA, USA) supplemented with 2% B-27, 20 ng/mL of epidermal growth factor (EGF, 78,006.1, STEMCELL Technologies, Vancouver, BC, Canada), and 20 ng/mL of fibroblast growth factor (FGF)-2 (78,003.1, STEMCELL Technologies) as mentioned previously [45]. A549 human adenocarcinoma lung epithelial cells were purchased from ATCC (CCL-185™) and were cultured in Gibco DMEM high glucose media (Thermo Fisher Scientific, Waltham, MA, USA) with 10% fetal bovine serum (FBS). G401 kidney rhabdoid tumor cells were also purchased from ATCC (CRL-1441™) and were cultured in McCoy's 5A Medium (ATCC, 30–2007™) supplemented with 10% FBS. SF8628 Human DIPG H3.3-K27 M cell line (Sigma-Aldrich, Cat# SCC127) was grown according to the supplier's protocol. All cell lines were cultured at 37 °C and 5% CO_2_ in an incubator.

### 2.2 Differentiation of human iPSCs into NK cells

**Fig. 1:**
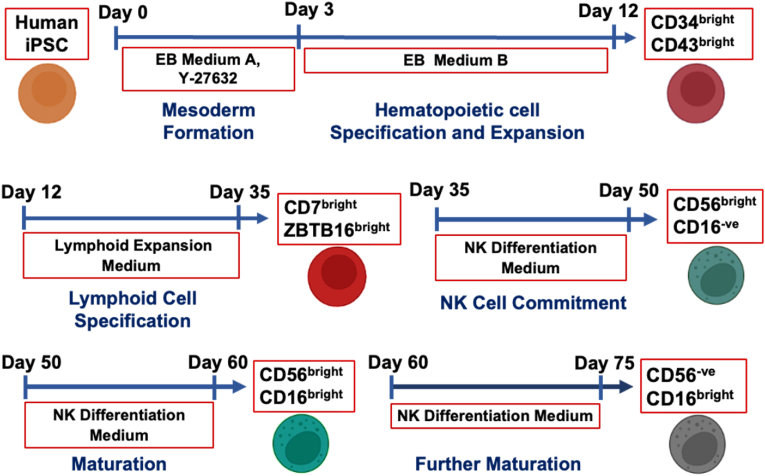
**Schematic representation of human iPSC differentiation into Natural Killer cells in five stages.** Hematopoietic progenitor differentiation is achieved using EB Medium A and B, characterized by CD34^bright^ CD43^bright^ phenotype. Subsequent progression to the lymphoid progenitor stage is marked by ZBTB16^bright^ CD7^bright^ expression, facilitated by Lymphoid Progenitor Expansion Medium (SFEMII) with expansion supplement. NK differentiation involves the use of an NK Differentiation Medium comprising SFEMII, NK Supplement, and UM729. The commitment to distinct NK cell subtypes includes CD56^bright^ CD16^−ve^, CD56^bright^ CD16^bright^, and further maturation into CD56^−ve^ CD16^bright^ NK cells. Abbreviations: iPSC, induced pluripotent stem cell; EB, embryonic body; SFEMII, serum-free expansion medium II; UM729, pyrimido-[4,5-*b*]-indole derivative.

The differentiation stages of human iPSCs into NK cells were achieved using reagents from the StemSpan NK Cell generation kit (Stem cell technologies). The NK cell differentiation protocol provided by the kit manufacturer was taken as a guide to obtain three phenotypes of NK cells: CD56^bright^ CD16^−ve^, CD56^bright^ CD16^bright^, and CD56^−ve^ CD16^bright^ NK cells. To initiate the feeder free-differentiation [46,47], the human iPSCs were seeded in the Ultra-Low Attachment (ULA) 24-well plates (Corning Inc., Corning, NY) at 3 × 10^5^ cells/well in differentiation medium composed of STEMdiff™ Hematopoietic EB Basal Medium with Supplement A (5 μL/mL) in the presence of ROCK inhibitor Y27632 (10 μM). On day 3, the media was replaced with STEMdiff™ Hematopoietic EB Basal Medium with Supplement B (100 μL/mL). The culture was incubated for 3 days at 37 °C with the half media change within 2 days. On day 6, the EBs were dissociated with Accumax (Stem cell technologies) and replated at the density of 1 × 10^5^ cells/well on a non-tissue culture-treated 6-well plate for another 6 days. Half of the media B was replaced with fresh media every two days till day 12. At this stage, the cells were studied for the expression of markers associated with hematopoiesis such as CD34 and CD43. On day 12, the progenitor cells were directed toward the lymphocyte lineage, where the cells were harvested, dissociated with Accutase (Stem cell technologies), and replated 1 × 10^5^ cells/well on a tissue culture-treated 6-well plate pre-coated with Matrigel. The lymphocyte differentiation media was prepared by mixing StemSpan™ SFEM II medium with StemSpan™ Lymphoid Progenitor Expansion Supplement (10X). The lymphocyte differentiation media was added to each well of 6-well plate and incubated at 37 °C. Half of the media was replaced with fresh media every 2–3 days, depending on the rate of cell proliferation. Upon confluency, the cells were detached with Accutase and replated on a T-25 flask (6 × 10^5^ cells/well) coated with the Matrigel and were expanded till day 30. Upon lymphoid progenitor differentiation and expansion, the cells were analyzed for the progenitor and lymphoid markers such as CD5, CD7, ZBTB16, and others. After 16 days of lymphoid expansion, the cells were further cultured for the commitment toward NK cells using differentiation medium (StemSpan™ SFEM II medium and StemSpan™ NK Cell Differentiation Supplement (100X)) and UM729. The UM729 is a pyrimido-[4,5-*b*]-indole derivative, which enhances the self-renewal of human hematopoietic stem cells during the differentiation to NK cells [16,48]. For the NK cell commitment, the Lymphoid progenitors were replated on a non-treated 6-well plate at 1 × 10^5^ cells/well and cultured in the NK cell differentiation media with the supplement and UM729 (1 μM). The media was changed every 2–3 days, depending on the cell density. On day 50, the cells detached with Accutase and analyzed for the expression of NK cell-specific markers such as CD56 and CD16. The cells initially obtained CD56^bright^ CD16^−ve^ immature phenotype on day 50, but with further differentiation, they matured into CD56^bright^ CD16^bright^ phenotype. Further maturation led to CD56^−ve^ CD16^bright^ NK cells. The schematic diagram of the differentiation protocol is shown in Fig. 1.

**Fig. 2:**
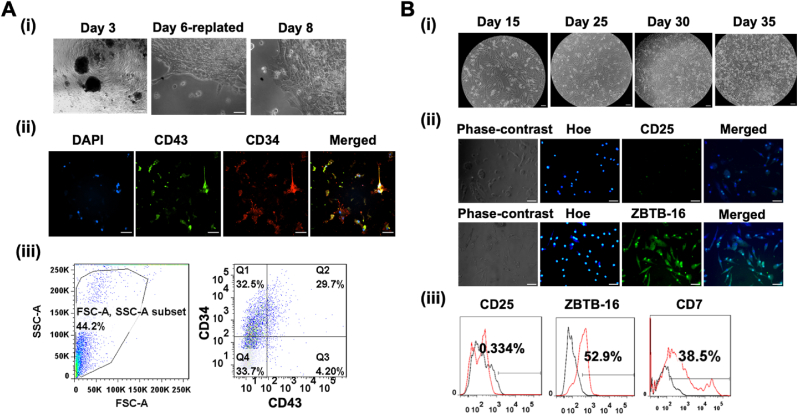
**Differentiation of hematopoietic and lymphoid progenitors from human iPSCs. (A)** (i) phase-contrast images captured on Day 3, Day 6, and Day 8 show the progression of myeloid progenitors, while (ii) qualitative analysis of CD34 and CD43 expression is conducted through immunocytochemistry (ICC), and (iii) flow cytometry provides a quantitative assessment of co-staining of CD34 and CD43. (B) (i) phase-contrast images tracking the development of lymphoid progenitors on Day 15, Day 20, Day 25, Day 30, and Day 35. Additionally, (ii) immunocytochemistry detects the expression of ZBTB16 and CD25 markers on Day 35, and (iii) flow cytometry quantifies ZBTB16, CD25, and CD7 on the same day of differentiation. ZBTB-16, Zinc finger and BTB domain containing 16; CD, Cluster of differentiation; Hoe, Hoechst 33,342. The scale bars in (A) and (B) represent 100 μm, corresponding to the magnification of 20x for ICC images and 10x for the phase contrast images.

**Fig. 3:**
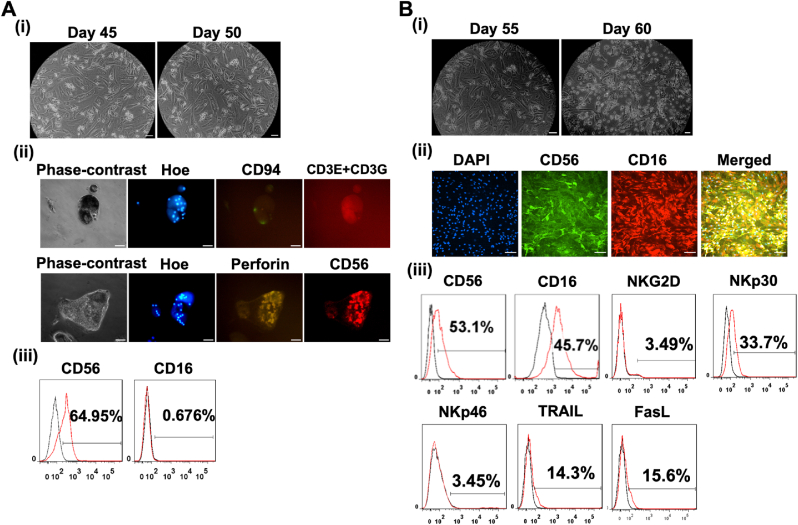
**The commitment of lymphoid progenitors to the CD56**^**bright**^**CD16**^**-ve**^**phenotype and their subsequent maturation into CD56**^**bright**^**CD16**^**bright**^**Natural Killer (NK) cells.** (A) (i) phase-contrast images taken on Day 45 and Day 50 reveal the differentiation of NK cells, while (ii) immunocytochemistry images demonstrate positive expression of the CD56 and negative for CD3E + CD3G, CD94, and perforin. (iii) Flow cytometry quantification of CD56 and CD16 indicates the commitment of lymphoid progenitors to premature NK cells. Two independent experiments verified the data, as shown in Supplementary Figure S1. (B) Further differentiation results in an intermediary maturation stage (CD56^bright^ CD16^bright^). (i) Phase-contrast images on Day 55 and Day 60 show continued differentiation. (ii) Co-staining immunocytochemistry images and (iii) flow cytometry quantification demonstrates positive expression of CD56 and CD16 markers, signifying the progression toward mature NK cells. Additionally, examination of NKG2D, NKp30, NKp46, TRAIL, and FasL at this stage was conducted through flow cytometry. NKG2D, NKG2-D type II integral membrane protein; NKp30, Natural killer cell p30-related protein; NKp46, Natural killer cell p46-related protein; TRAIL. The scale bar in (A) and (B) is 100 μm, corresponding to a magnification of 20x for ICC images and 10x for phase-contrast images.

### 2.3 Calcein-AM release assay to measure cell lysis

The differentiated iPSC-NK cells were cocultured with CHLA-02-ATRT and CHLA-05-ATRT tumor cell lines at various effector-to-target (E:T) ratios before measuring the cytotoxicity of NK cells via Calcein-AM-based assay. The target cells were plated at the density of 10,000 cells/well in the 96-well plates and were incubated at 37 °C for 30 min with 15 μM of Calcein-AM (Life Technologies C3099). Calcein-AM-labeled cell lines were cocultured with NK cells in a surface-treated 96-well plate for 4 h at 37 °C at three different E:T ratios (1:1, 3:1, and 6:1). For the measurement of spontaneous release, all target cells were incubated without NK cells. The total release was achieved by adding 4% Triton™ X-100 (Sigma-Aldrich) to the target cells. After the incubation, the supernatant was collected and transferred to a 96-well plate to measure the Calcein-AM release at 485 nm using the spectrophotometer (SpectraMax iD5). The percentage of specific lysis was calculated according to the following formula:

$$\left[\left[\left[\left(\text{Test}\;\text{release}\right)\;-\;\left(\text{Medium}\;\text{fluorescence}\right)\right]\;-\;\left[\left(\text{Spontaneous}\;\text{release}\right)\;-\;\left(\text{Medium}\;\text{fluorescence}\right)\right]\right]/\left[\left[\left(\text{Total}\;\text{release}\right)\;-\;\left(\text{Triton}\;X100\;\text{fluorescence}\right)\right]\;-\;\left[\left(\text{Spontaneous}\;\text{release}\right)\;-\;\left(\text{Medium}\;\text{fluorescence}\right)\right]\right]\;\right]\times 100$$

The spontaneous release was used as the negative control, whereas Triton X-100 fluorescence was used as the positive control.

### 2.4 3-(4,5-Dimethylthiazol-2-yl)-2,5-diphenyltetrazolium bromide (MTT) assay

The iPSC-NK cells were cocultured in a 96-well plate with ATRT cells at various E:T ratios for 4 h. After coculture, the effector cells in suspension were removed, and attached target cells were washed with phosphate-buffered saline (PBS). MTT stock solution (5 mg/mL) was diluted in cell culture media at 1:10 and was added to 96-well plates containing tumor cells. The culture was incubated for 30 min at 37 °C, and the cells were checked every 10 min till they turned purple. Once the cells turned purple, the supernatant was discarded, and the cells were washed with PBS. The formazan crystals were hydrolyzed by adding DMSO, and the absorbance of dissolved formazan was measured at 550 and 590 nm using a spectrophotometer. ATRT cells not exposed to iPSC-NK cells were used as negative controls, while cells treated with Triton X-100 were used as positive controls. The cell viability and relative percentage cell lysis data were calculated using the following formulae:

Relativepercentagecellviability(Y)=((viabilityoftargetcellsexposedtoNKcells)/(viabilityofunexposedtargetcells)x100);Relativepercentagecelllysis=100−Y

### 2.5 Immunocytochemistry (ICC)

The cells were plated on the wells of a Matrigel-coated 96-well plate at the density of 8000 cells/well for 24 h. The cells were then fixed with 10% neutral buffered formalin (VWR) for 45 min at room temperature (RT). After the fixation, for intracellular markers, the cells were permeabilized with 0.5% Triton X-100 for 10 min. To avoid non-specific bindings, the cells were blocked with a blocking buffer (2% fetal bovine serum in PBS) for 45 min. For each marker, primary antibody (Supplementary Tables S1 and S2) staining was performed overnight at 4 °C. For the secondary staining, Alexa Fluor 488 goat anti-mouse IgG and IgM or Alexa Fluor 594 goat anti-rabbit IgG (H + L) (Life Technologies) were used. Hoechst 33,342 (blue) was used to stain nuclei. A fluorescent microscope (Olympus IX70, Melville, NY) was used to take immunofluorescent images. Captured images were analyzed using ImageJ software (http://rsb.info.nih.gov/ij).

### 2.6 Intra- and extracellular marker quantification using flow cytometry

The cells were detached using Accumax and fixed using 10% neutral buffered formalin (VWR) for 45 min at RT and then washed 2–3 times with PBS. For intracellular markers, the cells were permeabilized with 100% cold methanol for 10 min. The cells were then blocked with a Blocking buffer (2% FBS in PBS) at RT for 45 min. The primary antibodies (Supplementary Table S2) were added to all single- and double-stained cell samples and incubated overnight at 4 °C. After 2–3 PBS washes, the secondary antibody Alexa Fluor 488 goat anti-mouse IgG and IgM, Alexa Fluor 568 goat anti-mouse IgG, or Alexa Fluor 594 goat anti-Rabbit IgG (H + L) (Life Technologies) were added and incubated for 1 h at RT. After 2–3 washes, the samples were run through a BD FACSCanto™ II flow cytometer (Becton Dickinson). While running the double-stained samples the compensation feature of the instrument was used to minimize the spectral overlap. The results were then analyzed against isotype controls using FlowJo software.

### 2.7 Reverse transcription-polymerase chain reaction (RT-PCR)

Total mRNA was extracted from iPSC-NK cells using RNeasy® Mini Kit (Qiagen, Valencia, CA). The isolated RNA was concentrated and cleaned using the DNA-Free RNA Kit (Zymo, Irvine, CA). About 1 μg of total RNA was used for reversed transcription. The RNA was anchored oligo-dT primers and treated with Superscript™ III (Invitrogen, Carlsbad, CA). The Primer-BLAST tool (https://www.ncbi.nlm.nih.gov/tools/primer-blast/) was used to design the primers (Supplementary Table S3). The melting points of these primers were checked using NetPrimer (https://www.premierbiosoft.com/netprimer/). The β-actin gene was used as an endogenous control and for the normalization of expression levels of all the other markers. The RT-PCR experiment was done using an ABI7500 instrument (Applied Biosystems, Foster City, CA) and SYBR Green PCR Master Mix (QuantaBio, Beverly, MA). The amplification was done as follows: 95 °C (10 min), 40 cycles of 95 °C (15 s), 55 °C (30 s), and 68 °C (30 s). The C_t_ values of the target genes were normalized to the C_t_ values of the endogenous β-actin control. The corrected C_t_ values were then compared for the IL15-exposed iPSC-NK and unexposed iPSC-NK cells. Fold changes in gene expression were calculated using the comparative C_t_ method: 2−^(ΔCt sample−ΔCt control)^ to obtain relative expression levels.

### 2.8 Live/dead quantification via flow cytometry

The Live/Dead™ staining kit (Molecular Probes) was used to measure live/dead cells. The ATRT cells were cocultured with NK cells (both interleukins-exposed and unexposed) for 7 h in the coculture media (1:1 ratio of DMEM/F-12 supplemented with 2% B-27, 20 ng/mL of epithelial growth factor (EGF) and 20 ng/mL of fibroblast growth factor (FGF)-2: RPMI media with 10% FBS). After coculture, ATRT cells were washed twice with PBS and suspended in Accumax for the formation of a single-cell suspension. The cells were incubated in DMEM/F-12 containing 5 μM Calcein-AM (green) and 4 μM ethidium homodimer I (red) in the dark for 20–25 min at RT. The samples were then run using BD FACSCanto™ II flow cytometer (Becton Dickinson) and analyzed against compensation controls using FlowJo software.

### 2.9 Degranulation and cytokine expression

iPSC-NK cells were cocultured with CHLA-02-ATRT and CHLA-05-ATRT target cells at the E:T ratio of 2:1 in a 24-well plate for 4 h at 37 °C. The cell surface expression of the degranulation marker CD107a was measured using ICC and flow cytometry following the published protocols [49,50]. For the measurement of intracellular cytokine expression in iPSC-NK cells, the cells were also cocultured with the ATRT cells for 4 h at 37 °C at the same E:T ratio. The expression of cytokines TNF-α and IL-6 was captured using ICC. The IFN- γ expression was quantified using flow cytometry.

### 2.10 ELISA for cytokine release measurement

The secretion of TNF-α, IL-6, and IFN-γ was measured through ELISA immune assays. The iPSC-NK cells were plated in the 96-well plate at the density of (1 × 10^5^ cells/mL) overnight. The next day, the iPSC-NK cells were exposed to ILs, CHLA-02-ATRT (E:T = 2:1), CHLA-05-ATRT (E:T = 2:1) for another 12 h. The unexposed condition was also cultured for the same period. The consumed media was taken out and centrifuged down at 400×*g* for 5 min. Then from each condition, 100 μL of supernatant was taken out for ELISA assay. The samples (Unexposed, IL-exposed, CHLA-02-ATRT exposed, and CHLA-05-ATRT exposed) and the standards were added to the pre-coated 96-well plate, and the assay was performed using the ELISA kit manufacturer's protocol. The ELISA assay for IL-6 and IFN-γ was performed using the protocol and the kit from BioLegend. For TNF-α, the ELISA protocol and kit from R&D Systems was used.

### 2.11 Statistical analysis

Each experiment was carried out at least two times with triplicates. The representative experiments were presented, and the results were expressed as [mean ± standard deviation]. To measure the statistical significance, one-way ANOVA followed by Fisher's LSD post hoc tests were performed. A *p*-value <0.05 was considered statistically significant.

## 3 Results

### 3.1 Feeder-free differentiation of human iPSCs yielded distinct maturational stages of NK cells

Human Epi-iPSCs were differentiated into three major NK cell subtypes that are commonly seen in human peripheral blood, i.e., CD56^bright^ CD16^−ve^, CD56^bright^ CD16^bright^, and CD56^−ve^ CD16^bright^ (Fig. 1) [6,51]. The feeder-free sequential differentiation process was performed in five stages. In stage I, CD34^bright^ and CD43^bright^ hematopoietic progenitors were obtained from iPSCs around day 12. At the end of stage II, cells expressed markers of innate lymphoid progenitors such as ZBTB-16 (Zinc finger and BTB domain-containing protein-16) around day 35, indicating innate lymphoid cell fate decision [52,53]. Once the progenitors showed lineage specification into innate immune cells, NK differentiation media was used to attain immature phenotype (CD56^bright^ CD16^−ve^) of NK cells around day 50, displaying their commitment to the NK cell lineage after stage III. CD56^bright^ CD16^−ve^ NK cells were further differentiated and matured into CD56^bright^ CD16^bright^ subtype (maturation stage 1) around day 60 (end of stage IV). Additional maturation (stage V) of the derived cells to day 75 gave rise to CD56^−ve^ CD16^bright^ cells (maturation stage 2) (Fig. 1).

The morphological changes of the cells were monitored throughout the differentiation. The human Epi-iPSCs aggregated to form embryoid bodies (EBs) on day 3. On day 6, the EBs were replated on tissue culture-treated plates and proliferated as hematopoietic progenitor cells. The morphologies on day 3, day 6, and day 8 are shown in Fig. 2Ai. CD34 and CD43 are cell surface markers of hematopoietic progenitors [54]. The immunocytochemistry results shown in Fig. 2Aii are positive for CD34 and CD43, indicating successful induction of iPSCs toward hematopoietic progenitors. To validate the results, 12-day progenitors were analyzed by flow cytometry. 29.7% of the cells co-expressed CD34 and CD43 (Fig. 2Aiii), and the percentage of CD34^bright^ and CD43 ^bright^ cells varied between 41.1-54.1% and 73.3–76.7%, respectively (Supplementary Fig. S1A). The results indicate the achievement of hematopoietic progenitor phenotype by the cells with the potential to commit to lymphoid precursors. [55].

The next stage of differentiation was lymphoid progenitor formation. The cells were replated on Matrigel to support lymphoid lineage induction and expansion. The morphology of the cells was captured every five days starting from day 15 (Fig. 2Bi). At this stage of differentiation, cells have low expression of CD25 [56] but high expression of ZBTB-16 and CD7. A high expression of ZBTB-16 indicates the development of innate lymphoid lineages [57], which was confirmed by the fluorescence imaging data shown in Fig. 2Bii. The percentage expression of CD25, ZBTB-16, and CD7 markers in lymphoid precursors on day 33 was quantified by flow cytometry as 0.33%, 52.9%, and 38.5%, respectively (Fig. 2Biii).

The third stage of differentiation involves the commitment of lymphoid cells to NK cells. CD56 is a well-known marker specific to NK cells, and its co-expression with CD16 signifies whether the differentiated cells are immunoregulatory or cytotoxic [58]. By day 50, the cells had successfully committed toward NK fate (Fig. 3Ai). The immunofluorescent images showed high expression of CD56 but negative results for CD94 and CD3E + CD3G (Fig. 3Aii)^.^ Flow cytometry results confirmed high CD56 expression (64.95–66.8%) and low CD16 expression (0.67–1.22%) (Fig. 3Aiii and Supplementary Fig. S1B). These results indicated the lineage specification of lymphoid progenitors toward the CD56^bright^ CD16^−ve^ subset of NK cells. The NK cells in this subset are immature with immunomodulatory phenotype [58,59]. The immature subset of NK cells has been shown to have low cytotoxicity; therefore, they do not secret perforins [49] and are negative for CD3 and CD94 markers [60,61].

These NK cells were further cultured in the NK differentiation media to achieve functionally mature CD56^bright^ CD16^bright^ NK cells (Fig. 3Bi). The CD16-expressing NK cells have shown a higher ability to kill cancer cells [58,62]. The immunofluorescence results (Fig. 3Bii) indicated the positive expression of CD56 and CD16 markers, which was followed by flow cytometric quantification of cells that express CD56(53.1%) and CD16 (45.7%) (Fig. 3Biii and Supplementary Fig. S2A). Markers such as CD7, CD5, GATA3, and CD3D **(**Supplementary Fig. S5**)** were also positive, which indicates the presence of some lymphoid progenitors or NKT populations. Cytometric data were also obtained for other general NK markers (Fig. 3Biii) - NKG2D (3.49%), NKp30 (33.7%), NKp46 (3.45%), TRAIL (14.3%), and FasL (15.6%). Together, these results revealed the maturation of CD56^bright^ CD16^−ve^ cells toward CD56^bright^ CD16^bright^ subset of NK cells.

### 3.2 Exposure to ATRT cells and IL-15 upregulated activation markers in and cytokine secretion by CD56^bright^ CD16^bright^ iPSC-NK cells

#### 3.2.1 Activation using ATRT cells

**Fig. 4:**
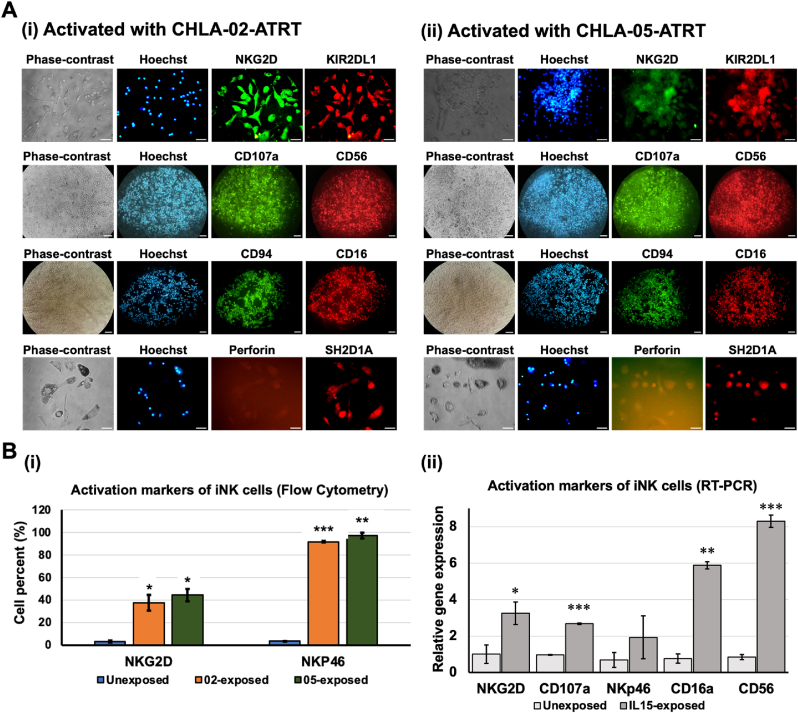
**Activation markers of CD56**^**bright**^**CD16**^**bright**^**iPSC-NK cells.** (A) The Immunocytochemistry data showed positive expression of the activation markers such as SH2D1A, NKG2D, CD107a, and inhibitory markers, KIR2DL and CD94 upon coculture with (i) CHLA-02-ATRT or (ii) CHLA-05-ATRT. (B) (i) The percentage expression of the activation markers, NKG2D and NKp46, in iPSC-NK cells before and after coculture was performed using flow cytometry. (ii) The relative gene expression of NK cell markers, including NKG2D, CD107a, NKp46, CD16a and CD56 after IL-15 treatment was also measured. KIR2DL1, killer cell immunoglobulin-like receptor 2DL1; SH2D1A, SH2 domain-containing protein 1A; NKG2D, NKG2-D type II integral membrane protein. The scale bar in (A) represents 100 μm, which corresponds to a 20x magnification. The statistically significant data are represented with “*”, “**”, and “***” for *p-*values <0.05, <0.01, and <0.001, respectively.

The iPSC-NK cells at maturation stage 1 (CD56^bright^ CD16^bright^) were grown with CHLA-02-ATRT and CHLA-05-ATRT cells and the expression of various activation markers was measured, such as NKG2D [3] and SH2D1A (Src homology 2 domain-containing protein 1A) [63], inhibitory markers including KIR2DL1 [64] and CD94 [64], cytotoxicity marker CD107a [65] and perforin [66], and NK cell-specific makers CD56 and CD16. SH2D1A plays an important role in the activation of immune cells, including NK cells [63]. Moreover, CD94 and NKG2D work together to maintain the survival of NK cells [67]. The results (Fig. 4A) showed positive expression of these markers, suggesting the successful activation of NK cells. The expression of NKp46 and NKG2D activation markers in iPSC-NK cells was significantly upregulated after coculture with ATRT cells as measured via flow cytometry (Fig. 4Bi and Supplementary Figs. S2B and C). Cytometric data also confirmed the expression of the NKp30 costimulatory receptor on these activated cells (Supplementary Figs. S2B and C).

**Fig. 5:**
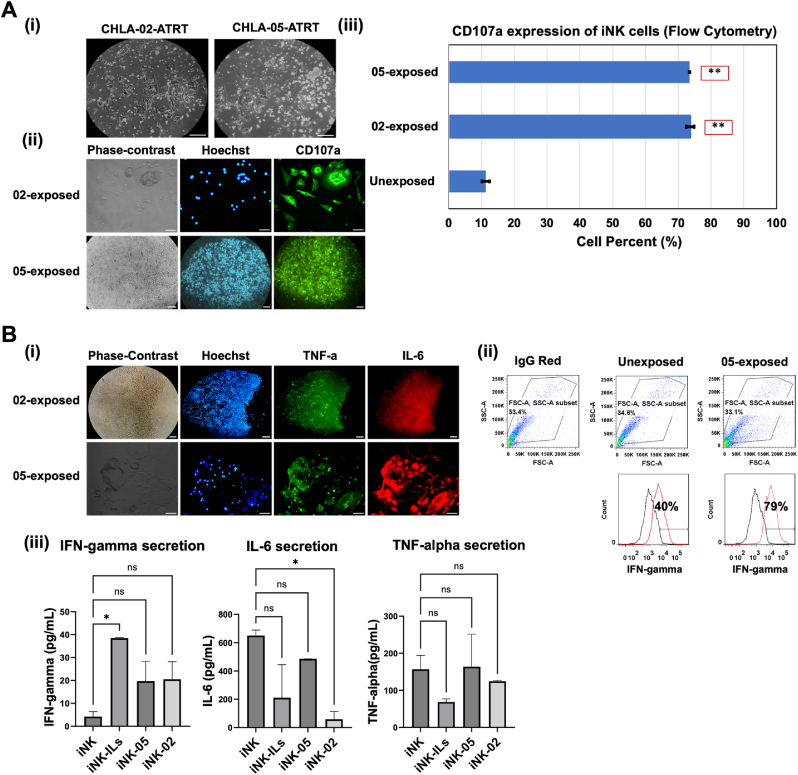
**Degranulation marker expression and cytokine production in CD56**^**bright**^**CD16**^**bright**^**iPSC-NK cells after exposure to ATRT cells.(A)** (i) Phase–contrast images showing coculture of iPSC-NK cells with CHLA-02-ATRT and CHLA-05-ATRT. (ii) Immunocytochemistry of degranulation marker CD107a in iPSC-NK cells after coculture with ATRT cells. (iii) Relative degranulation measurement in iPSC-NK cells before and after coculture with ATRT. “**”, *p-*value<0.01. **(B)** Cytokines production by human iPSC-NK cells. (i) Immunofluorescent images show positive TNF-alpha and IL-6 expression. (ii) iPSC-NK cells expressing IFN-gamma after exposure to CHLA-05-ATRT are quantified via flow cytometry. (iii) Cytokines released by iPSC-NK cells after exposure to interleukins, CHLA-05-ATRT, and CHLA-02-ATRT were quantified through ELISA assay. “*”, *p*-value<0.05. ns, non-significant difference. IL-6: Interleukin 6, TNF-a: Tumor necrosis factor alpha, IFN-gamma: Interferon-gamma. iNK:CD56^bright^ CD16^bright^ iPSC-derived NK cells, ILs: IL2, IL12, IL15, IL18, and IL21. Scale bar, 100 μm. The magnification is 20x for ICC images and 10x for phase-contrast images.

#### 3.2.2 Activation using IL-15

The role of interleukin (IL)-15 in the activation of human iPSC-NK cells was also investigated. The cells were exposed to IL-15 for 24 h, and NK cell-specific and activation markers were studied at mRNA levels. The results indicated that the expression of CD16a and CD56 mRNA was 6- and 8-fold higher upon exposure to IL-15 (Fig. 4Bii). Moreover, the activation markers NKG2D, CD107a, and NKp46 were also upregulated by 3-, 2.5-, and 2-fold, respectively (Fig. 4Bii).

The cytotoxic activity of NK cells can be measured by the CD107a/LAMP1 (lysosomal-associated membrane protein-1) marker on NK cell surface. This marker is associated with the release of lytic substances called perforin and granzymes from granules - a process called degranulation. This process activates the killing ability of NK cells [68], and CD107a/LAMP1 shows higher expression in activated NK cells during degranulation [65]. Immunofluorescent imaging of iPSC-NK cells after coculture with CHLA-02-ATRT and CHLA-05-ATRT cells (Fig. 5Ai) showed positive expression of the CD107a marker (Fig. 5Aii). The level of CD107a expression was compared before and after NK activation using flow cytometry. The results showed high expression (>70%) of CD107a/LAMP1 in the activated NK cells (Fig. 5Aiii), implying CD56^bright^ CD16^bright^ iPSC-NK cells can attack target ATRT cells through a degranulation mechanism.

Similarly, when NK cells get activated, they release cytokines, especially interferon (IFN)-γ. IFN-γ production potentially increases the Fas-ligand-based cytotoxicity of NK cells [69]. The expression of IL-6 and tumor necrosis factor (TNF)-α (Fig. 5Bi) was positive in ATRT-activated iPSC-NK cells. Flow cytometry results showed that the expression of IFN-γ increased significantly from 40% to 79% after exposure to tumor cells, suggesting one of the functional antitumor mechanisms of CD56^bright^ CD16^bright^ iPSC-NK cells (Fig. 5Bii). The quantification of cytokine release revealed an overall increase in IFN-γ secretion by activated NK cells compared to normal iPSC-NK; however, only interleukin-exposed (IL-2, IL-12, IL-15, IL-21, and IL-18) cells manifested a significant difference. Interestingly, IL-6 secretion tended to decrease in activated NK cells, with a noteworthy reduction observed in cells exposed to CHLA-02-ATRT (Fig. 5Biii). TNF-α secretion did not change significantly in activated NK cells compared to normal iPSC-NK cells. However, its release was the highest in the CHLA-05-ATRT-activated group compared to other activated groups.

### 3.3 CD56^bright^ CD16^bright^ iPSC-NK cells showed functional cytotoxicity against ATRT and other tumor cells

**Fig. 6:**
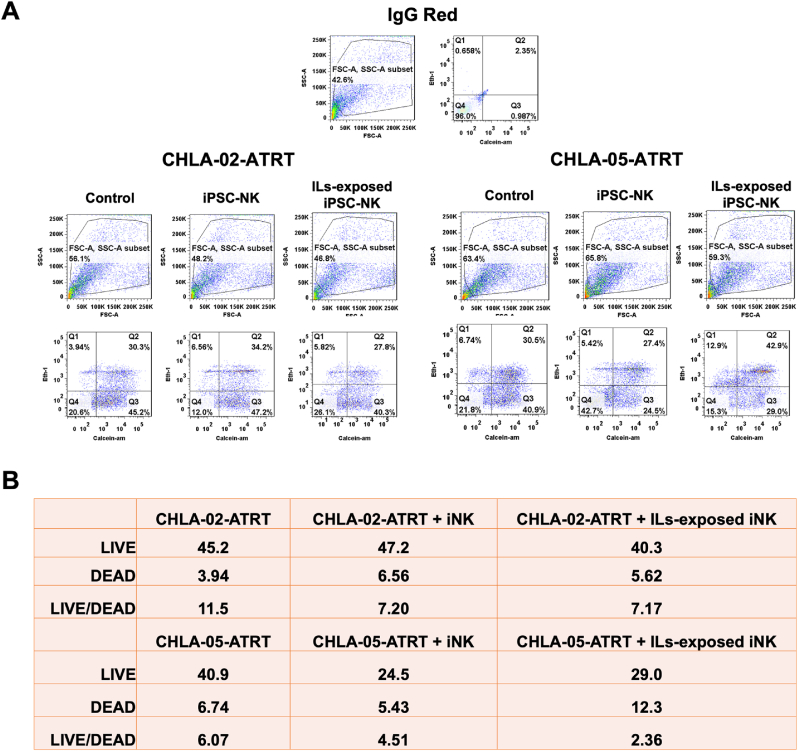
**The cytotoxicity analysis of interleukins (ILs)-exposed iPSC-NK cells against ATRT cells.** (A) Flow cytometry-based Calcein-AM assay. The quadrant plot displays the percentage expression of the live cell marker (Calcein-AM) on the x-axis and the dead cell marker (ETHD-1) on the y-axis for CHLA-02-ATRT or CHLA-05-ATRT after coculture with iPSC-NK cells. (B) The table depicts the percentage expression of live and dead cells as measured in (A). The live/dead ratio is observed to be lower in the coculture of both CHLA-02-ATRT and CHLA-05-ATRT with interleukin-exposed iPSC-NK cells compared to unexposed iPSC-NK cells. ILs: IL2, IL12, IL15, IL18, and IL21.

To test the anti-tumor activity, iPSC-NK cells were cocultured with ATRT cells at various effector-to-target ratios. Additionally, the effect of cytokine priming on cytotoxicity was also investigated by exposing iPSC-NK cells to various interleukins. Cytokines such as IL-2, IL-15, and IL-18 have previously been proven to enhance the cytotoxicity of NK cells [70]. IL-2, IL-15, and IL-12 increase the activation and cytotoxicity of NK cells by upregulating the function of TRAIL (TNF–related apoptosis-inducing ligand) and fas-ligand [71]. IL-2 and IL-12 have been shown to work together to induce cytotoxicity of NK cells by increasing their IFN-γ secretion [72]. Moreover, the combined exposure of IL-21, IL-15, and IL-18 enhanced the production of IFN-γ in NK cells [73]. This study tested the collective role of cytokines IL-2, IL-12, IL-15, IL-21, and IL-18 toward cytotoxicity of iPSC-NK cells against CHLA-02-ATRT and CHLA-05-ATRT. According to the results of live/dead flow cytometry (Fig. 6), the ratio of live to dead ATRT cells was 7.2 for CHLA-02-ATRT and 4.51 for CHLA-05-ATRT in coculture with IL-exposed iPSC-NK cells. The ratio was 7.17 for CHLA-02-ATRT and 2.36 for CHLA-05-ATRT in coculture with IL-unexposed iPSC-NK cells. These ratios were compared with the values of ATRT cells only, which were 11.5 for CHLA-02-ATRT and 6.07 for CHLA-05-ATRT. Hence, the ratio of live to dead ATRT cells was lower in coculture with IL-exposed iPSC-NK cells compared to IL-unexposed iPSC-NK cells.

**Fig. 7:**
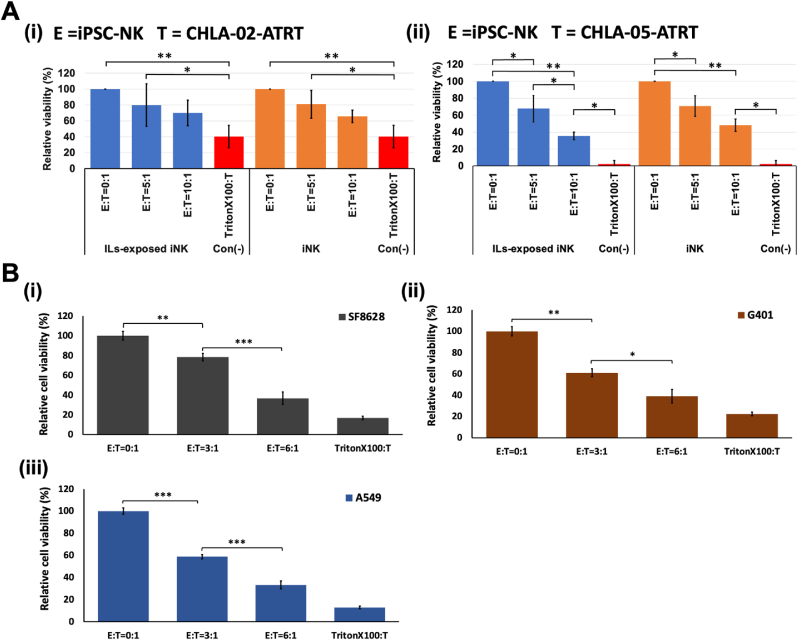
**Decrease in the viability of cancer cells upon exposure to CD56**^**bright**^**CD16**^**bright**^**iPSC-NK cells.** (A) Ratio-dependent decrease in CHLA-02-ATRT and CHLA-05-ATRT cell viability upon coculture with CD56^bright^ CD16^bright^ iPSC-NK cells; (B) Ratio-dependent decrease in the viability of other types of tumor cells upon coculture with CD56^bright^ CD16^bright^ iPSC-NK cells, including SF8628, a diffuse intrinsic pontine glioma cell line, G401, a kidney rhabdoid tumor cell line, and A549, a lung carcinoma cell line. iNK, CD56^bright^ CD16^bright^ iPSC-NK. Statistical significance is denoted by *, **, and *** for *p*-values less than 0.05, 0.01, and 0.001, respectively.

Additionally, the killing of ATRT cells by human iPSC-NK cells was investigated at multiple E:T ratios using MTT assay and Calcein AM cytotoxicity assay. MTT assay was performed to measure the relative cell viability of ATRT cells by the action of both IL-exposed and IL-unexposed iPSC-NK cells (Fig. 7A). The E:T ratios chosen to calculate the percentage of relative cell viability were 0:1 (negative control), 5:1, and 10:1, along with a positive control (4% Triton X-100). According to the results, a ratio-dependent decrease in cell viability of ATRT cells was observed. At the highest ratio (10:1), the percentage viability of ATRT cells by IL-exposed iPSC-NK cells was 70% for CHLA-02-ATRT and 36% for CHLA-05-ATRT. The percentage of cell viability was 66% (CHLA-02-ATRT) and 48% (CHLA-05-ATRT) for IL-unexposed iPSC-NK cells. At all ratios, the percentage viability of CHLA-05-ATRT was higher compared to CHLA-02-ATRT cells, indicating higher cytotoxic effects of iPSC-NK cells toward CHLA-05-ATRT cells. These results, along with relative cell death results (Supplementary Fig. S3), were consistent with the cytotoxic assay data in Fig. 7.

To compare the cytotoxic potential of iPSC-NK cells with other types of NK cells, NK92mi cells (ATCC, Cat#CRL-2408) were studied. NK92mi cells are derived from the peripheral blood of a non-Hodgkin's lymphoma patient [74,75] and have displayed cytolytic activity against renal cell cancer, multiple myeloma, and Hodgkin lymphoma [74,76,77] Therefore, these NK cells were used for the comparative cytotoxic analysis of iPSC -NK cells. The antitumor activity of NK92mi cells was tested in both ATRT cells - CHLA-02-ATRT and CHLA-05-ATRT (Supplementary Fig. S4). The percentages of the specific lysis of ATRT cells were determined using a Calcein AM-based cytotoxicity assay. The anti-tumor activity of iPSC-NK cells and NK92mi cells at various ratios was measured. For CHLA-02-ATRT, the percentage lysis by IL-exposed and unexposed iPSC-NK cells at the E:T ratio of 6:1 was 18% and 33%, respectively (Supplementary Fig. S4A). While the lysis of CHLA-02-ATRT by NK92mi cells at the E:T ratio of 4:1 was 11% and 13% percentage (Supplementary Fig. S4B). For CHLA-05-ATRT, the percentage lysis by IL-exposed and unexposed iPSC-NK cells at the same E:T ratio was 24% and 22%, respectively. Whereas the lysis of CHLA-05-ATRT by NK92mi cells at the E:T ratio of 4:1 was 20% and 19%, respectively. The specific cell lysis data indicated comparable cytolytic activity of both iPSC-NK and NK92mi cells at these ratios.

The anti-tumor activity of iPSC-NK cells was also tested in other tumor cells, i.e., SF8628 (DIPG), G401 and A549. Diffuse intrinsic pontine glioma (DIPG) is an aggressive tumor originates in the ventral pons of the brain stem [[78], [79]]. SF8628 is a DIPG cell line derived from H3·3K27 M DIPG patient [80]. Similarly, G401 cells are also malignant rhabdoid kidney tumor cells derived from a male child [81]. Along with these two pediatric cells, A549 (lung cancer) cells were used as targets to test the cytotoxic efficacy of iPSC-NK cells (Fig. 7B). The iPSC-NK cells were cocultured with each target at three ratios (E: T = 0:1, 3:1, and 6:1). The results indicated effective lysis of these tumor cells by iPSC-NK cells at both 3:1 and 6:1 ratio. At 3:1, A549 cells (58%) had the lowest viability compared to SF8628 (78%) and G401 (61%) (Fig. 7B), suggesting a greater sensitivity of the lung cancer cells toward iPSC-NK cells. At 6:1, the iPSC-NK cells were showing a similar cytolytic activity. However, at this ratio, SF8628 (36%) and G401 (39%) showed comparable viability (Fig. 7B–i and ii). The iPSC-NK cells were added in all cocultures at three ratios (E: T = 0:1, 3:1, and 6:1). The results indicated effective lysis of these tumor cells by iPSC-NK cells at these ratios.

Overall, the ATRT cells were the most aggressive compared to all studied pediatric and non-pediatric tumor cells; however, iPSC-NK cells were able to effectively lyse CHLA-05-ATRT cells. CHLA-02-ATRT cells displayed relatively more resistance to iPSC-NK cells. Among all target cells, the lung tumor cells (A549) were the most sensitive to the iPSC-NK cells. The rhabdoid tumor cells (G401) and glioma cells (SF8628), despite their malignant nature, were also sensitive to the cytotoxicity of iPSC-NK cells.

### 3.4 Further maturation of iPSC-NK cells into CD56^−ve^ CD16^bright^ phenotype abated cytotoxic potential

**Fig. 8:**
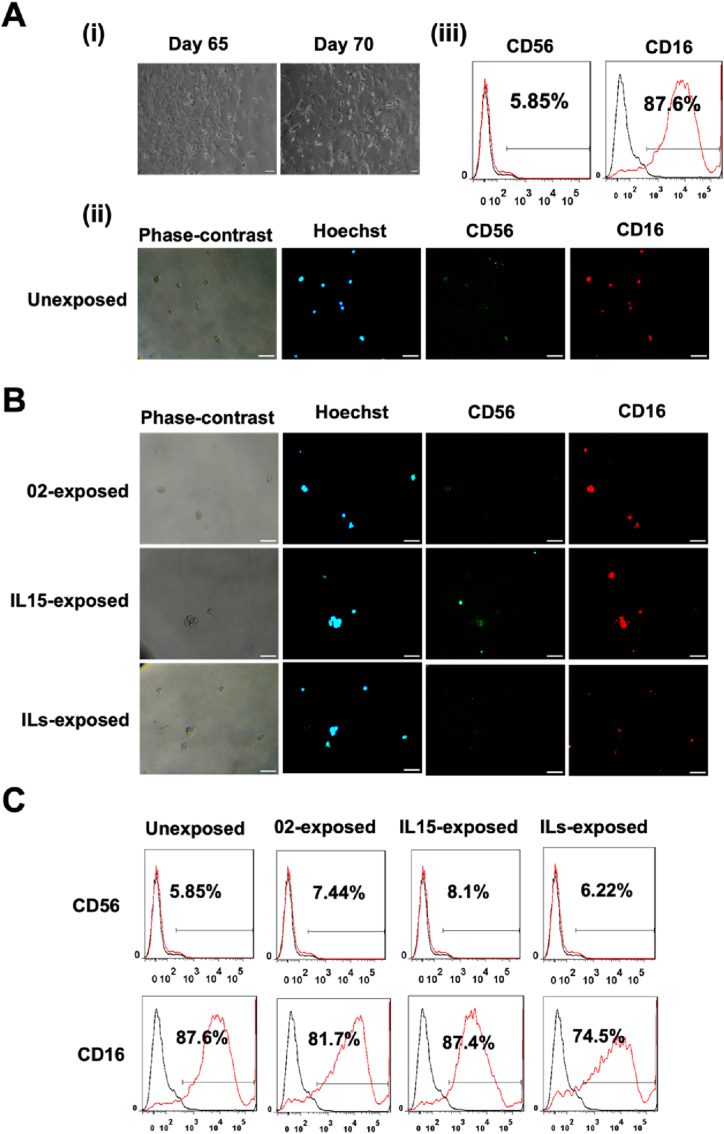
**Further maturation of iPSC-NK cells into CD56**^**-ve**^**CD16**^**bright**^**phenotype.** (A) (i) Phase-contrast imaging on Day 65 and Day 70. (ii) Immunocytochemistry (ICC) images of CD56 and CD16. (iii) Flow cytometry quantification shows the highly positive expression of CD16 and low expression for the CD56 marker. (B) Immunocytochemistry detects the expression of CD56 and CD16 in iPSC-NK cells after activation using CHLA-02-ATRT, IL-15, and a combination of interleukins (IL2, IL12, IL15, IL18, and IL21). (C) The percentage expression of CD16 and CD56 under each condition remains relatively stable. Scale bar: 100 μm.

Human iPSC-NK cells were further matured till day 75 (Fig. 8Ai), and the expression of CD56 and CD16 markers was measured. Negative expression of CD56 and positive expression of CD16 marker was observed (Fig. 8Aii). Additionally, the quantitative results showed that the percentage of CD16^+^ cells and CD56^+^ cells was around 5% and 87%, respectively (Fig. 8Aiii). These cells were exposed to IL-15, the combination of ILs (IL-2, IL-12, IL-15, IL-18, and IL-21), and CHLA-02-ATRT cell to reveal the effects of the exposures on the expression of phenotypic markers CD56 and CD16. The results indicated that the exposures did not have any significant effect on the phenotype of iPSC-NK cells (Fig. 8B and **C**).

**Fig. 9:**
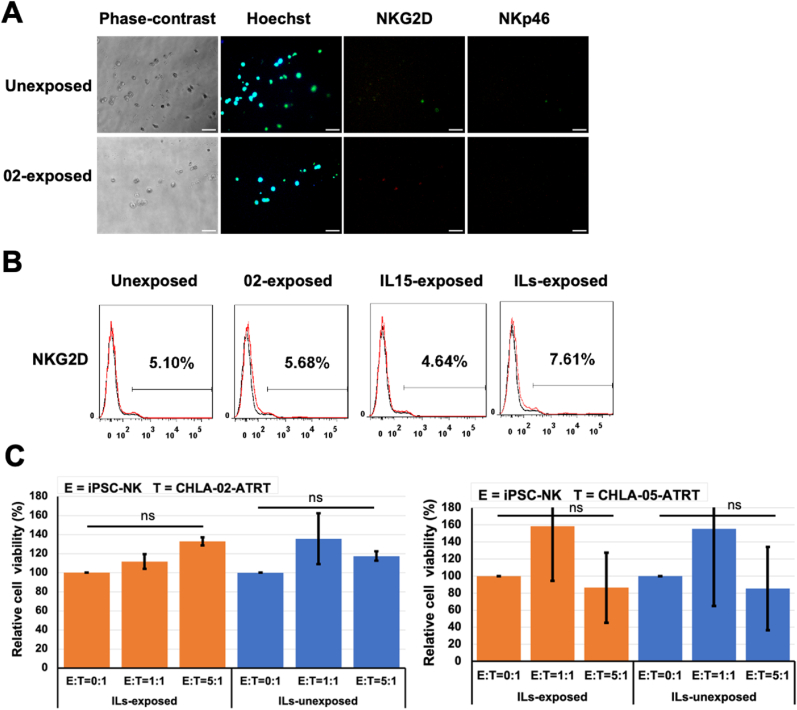
**Characterization of the activation markers in CD56**^**-ve**^**CD16**^**bright**^**iPSC-NK cells and their cytotoxicity toward ATRT cells.** (A–B) Immunocytochemistry and flow cytometry data indicate the absence of expression for NKG2D and NKp46 markers. At this stage, NKG2D in iPSC-NK cells was not upregulated after coculture with CHLA-02-ATRT. (C) Effects of ILs-exposed iPSC-NK cells versus ILs-unexposed iPSC-NK cells on cell viability of CHLA-02-ATRT and CHLA-05-ATRT cells have no statistically significant decrease in cell viability compared to the untreated group, E: T = 0:1. ns, non-significant difference. The data were verified by two independent experiments, and each experiment has triplicates (n = 3).

To assess the functionality of CD56^−ve^ CD16^bright^ iPSC-NK cells as effectors, the expression of various cell surface activation markers was determined, and the cytotoxicity of iPSC-NK cells was tested. The results indicated a negative expression of NKG2D and NKp46 activation markers (Fig. 9A and B). This suggested the loss of cytotoxic properties of NK cells associated with NKG2D and NKp46 in CD56^−ve^ CD16^bright^ cells. Additionally, the cytotoxicity test showed no change in cell viability of ATRT cells upon exposure to CD56^−ve^ CD16^bright^ iPSC-NK cells (Fig. 9C). The lack of functional properties of these iPSC-NK cells suggested possible senescence of the cells [10].

## 4 Discussion

The human iPSC-derived NK cells provide off-the-shelf immunotherapy and display effective antitumor activity in many *in vitro* and *in vivo* models [43]. This study evaluated various maturational stages of human NK cells during their feeder-free differentiation and maturation from human iPSCs. The demonstration of iPSCs differentiating into effector NK cells in feeder-free culture conditions forms a potential basis for a non-immunogenic and efficacious immunotherapeutic modality [46].

The first stage of differentiation is the hematopoietic progenitor stage, characterized based on the high expression of CD34 and CD43 markers [55]. The second stage is characterized by the ability of the progenitors to achieve lymphoid (CD7^bright^) [82] and innate immune cell lineage (ZBTB-16^bright^). ZBTB-16 is also known as promyelocytic leukemia zinc finger (PLZF), which enables lymphoid progenitors to attain innate immune cell properties [52] and was highly positive in innate immune cell progenitors. In the third stage, the lymphoid progenitors committed toward CD56^bright^ CD16^−ve^ phenotype of NK cells. This phenotype is known to have a longer telomere than the CD56^−ve^ subset [83]. Therefore, CD56^bright^ CD16^−ve^ cells are deemed to be an immature subset of NK cells [84,85] that lack cytotoxic function and exhibit immune regulatory properties [86].

The maturation of NK cells resulted in CD56^bright^ CD16^bright^ phenotype (stage IV), which is known to be a functionally active and cytotoxic subtype [87]. CD16, also known as Fc receptor γRIII, is associated with antibody-dependent cell-mediated cytotoxicity (ADCC) [88,89]. Therefore, the CD16^bright^ NK cell subtype can effectively attack cancer cells by engaging their Fc receptor (CD16) to bind the Fc portion of the mediator antibody that recognizes tumor-associated antigens [90]. The CD16^bright^ iPSC-NK cells are cytotoxic against different tumor cells, including human leukemia K562 cells [87]. Additionally, CD56^bright^ CD16^bright^ NK cells from the peripheral blood of donors displayed cytotoxic potential in patients with refractory acute myeloid leukemia [91]. Thus, obtaining CD56^bright^ CD16^bright^ NK cells from iPSCs for eliminating malignant cells could be a potential avenue in cancer management.

To elucidate the response of CD56^bright^ CD16^bright^ (stage IV) iPSC-NK cells to IL exposure, cells were exposed to IL-15 for 24 h. IL-15 was the choice of interleukin because it has previously been found to control tumor growth by improving the killing potential of NK cells and the proliferation of NK cells both *in vitro* and *in vivo* [92,93]. The exposure resulted in an upregulation of activation, cytolytic, and NK cell-specific makers in iPSC-NK cells, validating the attainment of effector role by these cells.

The CD56^bright^ CD16^bright^ NK cells showed higher expression of activation markers and release of cytokines IFN-γ and TNF-α upon exposure to ATRT cell lines. Previous findings suggest that the release of IFN-γ improves the cytolytic potential of NK cells [69] and reduces tumor metastasis [94]. Beyond these mechanisms, NK cells release lytic granules carrying perforin and granzyme B to attack their target [5], CD107a, which is a marker for the degranulation of NK cells, was also seen to be highly expressed by activated iPSC-NK cells. These findings cumulatively suggest the functional maturation of the derived NK cells and their capability to employ a repertoire of cytotoxic mechanisms post-activation.

The efficacy of CD56^bright^ CD16^bright^ iPSC-NK cells against the ATRT cell lines was tested. Cell viability and cell lysis data showed a higher killing of CHLA-05-ATRT compared to that of CHLA-02-ATRT cells by human iPSC-NK cells. The observed difference in the killing efficiency may be partly explained by varied immunogenicity and sensitivity to apoptosis signals across cancer cells [95]. However, all results showed the ratio-dependent cytotoxicity of human iPSC-NK cells toward both ATRT cell lines. The iPSC-NK cells also exhibited cytolytic functionality toward other aggressive tumor cells, such as glioma (SF8628) and kidney rhabdoid (G401) cells, as well as lung cancer (A549) cells. The anti-tumor activity of iPSC-NK cells among diverse tumor types *in vitro* indicates their potentiality in cell-based anticancer immunotherapy.

The CD56^bright^ CD16^bright^ phenotype of NK cells was allowed to mature further into the CD56^−ve^ CD16^bright^ subtype [96]. Cells of this subtype lacked the expression of NKG2D and NKp46 markers**,** implying their functional impairment. The CD56^−ve^ CD16^bright^ NK cells also did not show any significant cytotoxicity when exposed to ATRT cells. Such dysfunctionality of CD56^−ve^ CD16^bright^ NK cells may result from cell senescence and physiological aging [10,97].

This study provides a novel perspective of iPSC-NK cell derivation by identifying various phenotypes of NK cells at each stage of their development and maturation. Moreover, the cytotoxic potential of the identified mature phenotypes of NK cells was tested against rare pediatric tumors such as ATRT cells, which has not yet been reported. The derived NK cells displayed cytotoxic properties at stage IV of their differentiation, but a decline in those properties was observed upon further maturation.

Although there is no absolute cure for pediatric brain tumors, improved survival without cytokine release syndrome has been achieved with NK cell therapies for glioma, medulloblastoma [98], and high-risk malignant brain tumors [27]. In this study, the iPSC-NK cells showed cytotoxic efficacy toward various malignant tumor cells, including rare pediatric cancer cell lines. These *in vitro* cytotoxicity studies, however, can only provide a foundational understanding of their short-term functional properties. Investigating their post-infusion persistence, expansion capacity, and long-term efficacy in complex *in vivo* environments is required to advance the knowledge about their immune properties for therapeutic use, presenting a translational future research direction.

In summary, our work demonstrates the derivation of common NK cell subtypes from iPSCs, including the CD56^bright^ CD16^bright^ phenotype with *in vitro* effector function. The characterization of their cytotoxic properties, antitumor activity, and aging-induced functional decrement serves as the basis for testing the *in vivo* persistence and efficacy of NK cells. Nevertheless, additional studies are needed to address constraints regarding extended culture conditions and immature phenotype of iPSC-NK cells [99] so that NK cell therapy transitions from promise to pragmatism.

## 5 Conclusion

In this study, we performed a feeder-free differentiation of NK cells from iPSCs. We characterized various maturational subtypes of iPSC-NK cells and studied their phenotypic and functional properties. Mature iPSC-NK cells of CD56^bright^ CD16^bright^ phenotype expressed activation markers in response to interleukin stimuli and produced cytokines upon exposure to malignant brain rhabdoid tumor cells. Moreover, CD56^bright^ CD16^bright^ iPSC-NK cells demonstrated functional cytotoxicity by killing malignant cells. However, further maturation of the cells yielded a dysfunctional CD56^−ve^ CD16^bright^ subtype. The results suggest that iPSC-NK cells with effector properties have the potential to form the basis of an effective therapeutic strategy against aggressive pediatric brain tumors.

## Ethics approval and consent to participate

Not applicable.

## Data availability

The datasets generated and analyzed during the current study are present in the main text and supplementary file of this paper. Additional relevant data will be available from the corresponding authors on reasonable request.

.

## CRediT authorship contribution statement

**Sonia Kiran:** Writing – review & editing, Writing – original draft, Visualization, Methodology, Investigation, Formal analysis, Data curation. **Yu Xue:** Writing – review & editing, Visualization, Validation, Methodology, Investigation, Formal analysis, Data curation. **Drishty B. Sarker:** Writing – review & editing, Writing – original draft, Visualization, Validation, Methodology, Investigation, Formal analysis. **Yan Li:** Writing – review & editing, Supervision, Resources, Project administration, Methodology, Funding acquisition, Formal analysis. **Qing-Xiang Amy Sang:** Writing – review & editing, Writing – original draft, Visualization, Validation, Supervision, Resources, Project administration, Methodology, Investigation, Funding acquisition, Formal analysis, Conceptualization.

## Declaration of competing interest

None.
