# Supplementary material for: Feeder-free differentiation of human iPSCs into natural killer cells with cytotoxic potential against malignant brain rhabdoid tumor cells
Source: Bioact Mater. 2024 Mar 8;36:301–16. doi: 10.1016/j.bioactmat.2024.02.031 (PMC10940949; doi:10.1016/j.bioactmat.2024.02.031)
Supplement: Multimedia component 1 [file mmc1.docx]

**Supplementary Information**

**Feeder-free differentiation of human iPSCs into natural killer cells with cytotoxic potential against malignant brain rhabdoid tumor cells**

**Running title:** **Cytotoxicity of hiPSC-NK cells to cancer cells**

**Supplementary Figure S1. A repetition of flow cytometry for the reproducibility of the iPSC-NK cell differentiation protocol**. It showed relatively consistent results between batches 1 (i) and 2 (ii). **(A)** Hematopoietic progenitor markers, CD34 and CD43. **(B)** Immature stage, CD56^bright^ CD16^-ve^ iPSC-NK cells.

**
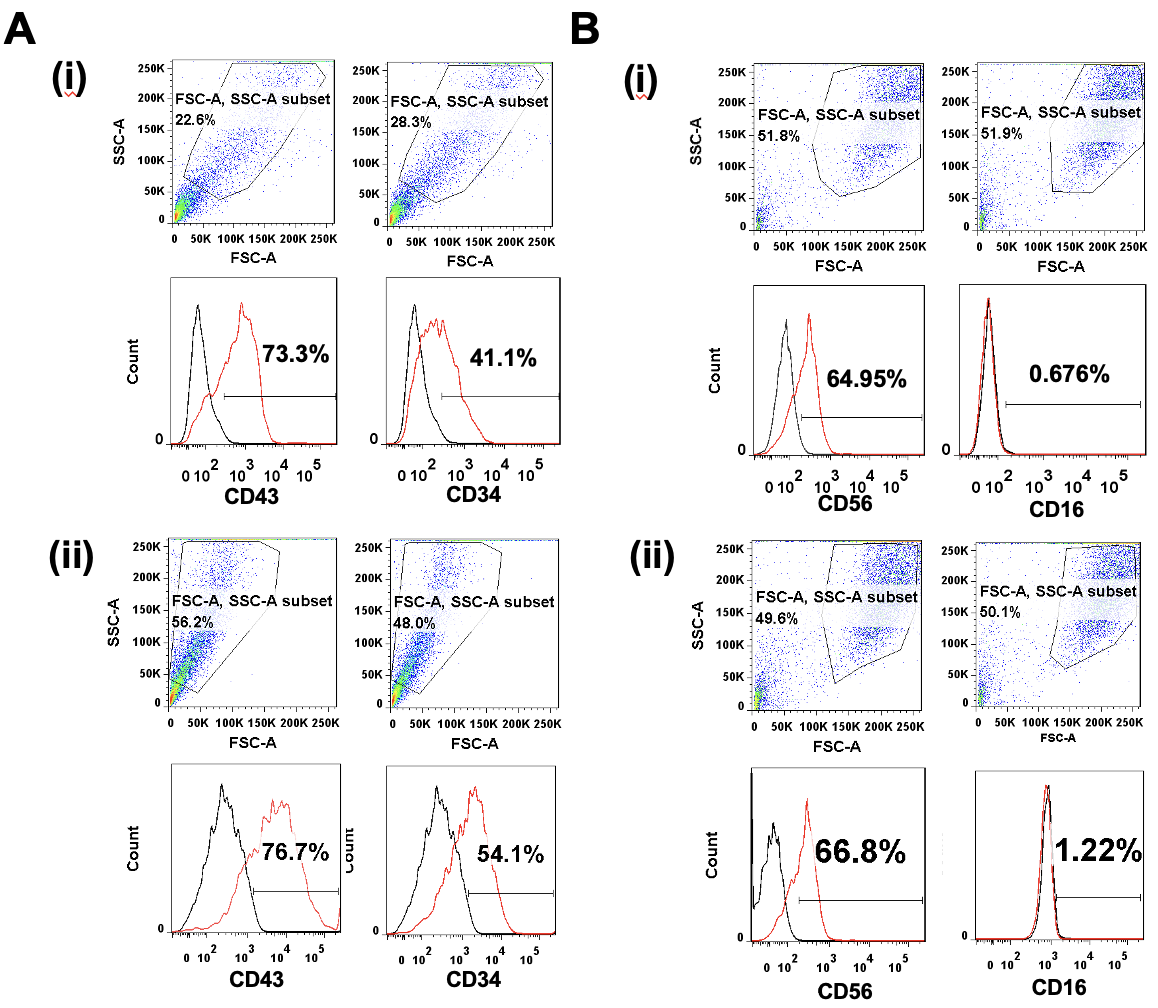
**

**Supplementary Figure S2. The differentiation stage of CD56^bright^ CD16^bright^ iPSC-NK cells: (A)**The gating events of CD56^bright^ CD16^bright^ iPSC-NK cells in Figure 3B(ii). **(B-C)** The percent expression of NKp46 (99.6%) and NKG2D (41%) in CD56^bright^ CD16^bright^ iPSC-NK cells after exposure to CHLA-05-ATRT cells were higher than NKp46 (91%) and NKG2D (30%) after exposure to CHLA-02-ATRT cells. However, NKp30 was expressed in the majority of iPSC-NK cells after exposure to either CHLA-02-ATRT or CHLA-05-ATRT. NKp46, Natural killer cell p46-related protein; NKG2D, NKG2-D type II integral membrane protein; NKp30, Natural killer cell p30-related protein.


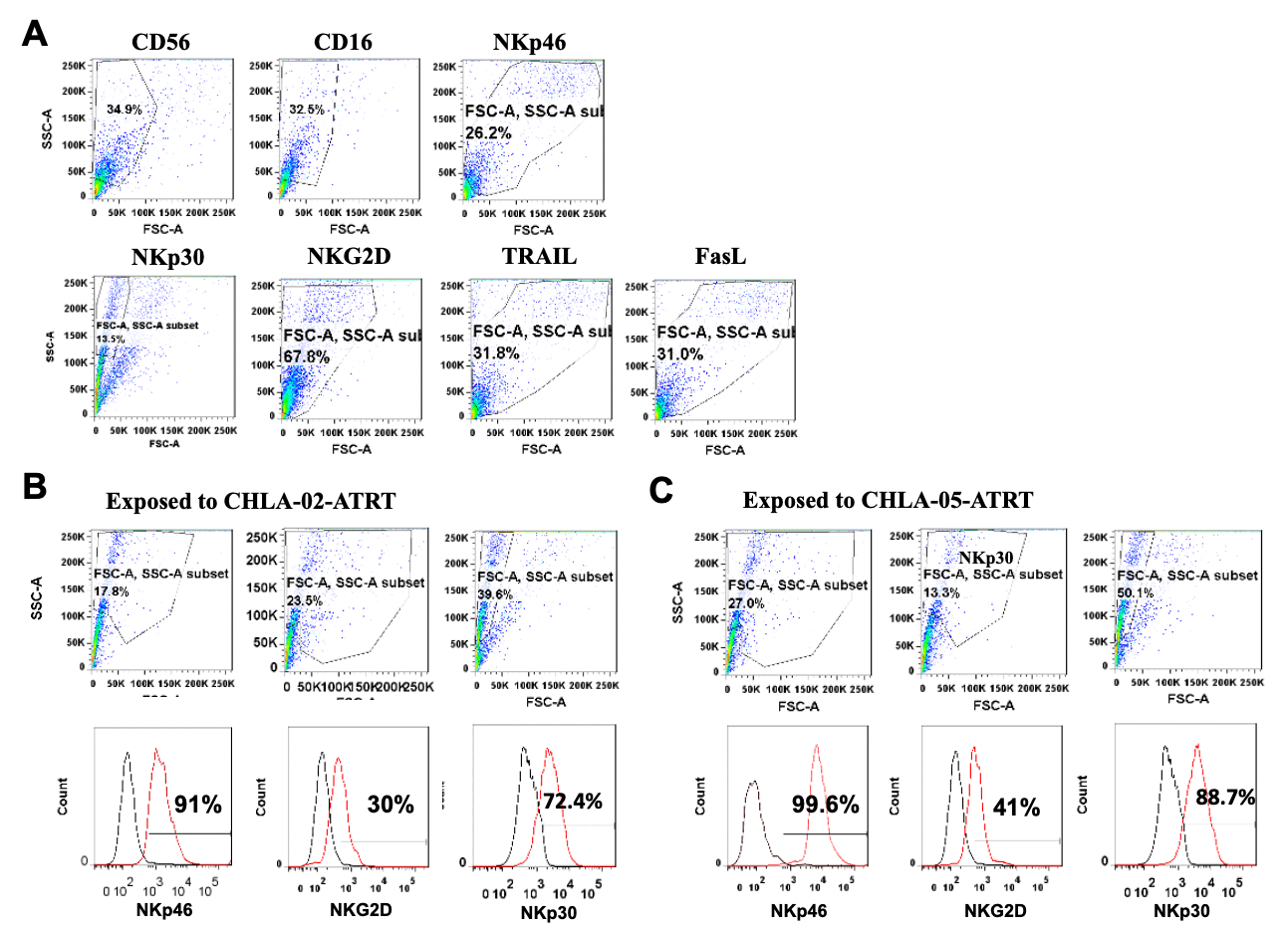


**Supplementary Figure S3: Assessing the efficacy of CD56^bright^ CD16^bright^ iPSC-NK cells in inducing cell death of ATRT cells.** The MTT assay was employed to quantify the relative cell death of ATRT cells mediated by iPSC-NK cells across various effector-to-target ratios (E:T). The percentage of cell death was normalized to the E:T condition of 0:1.


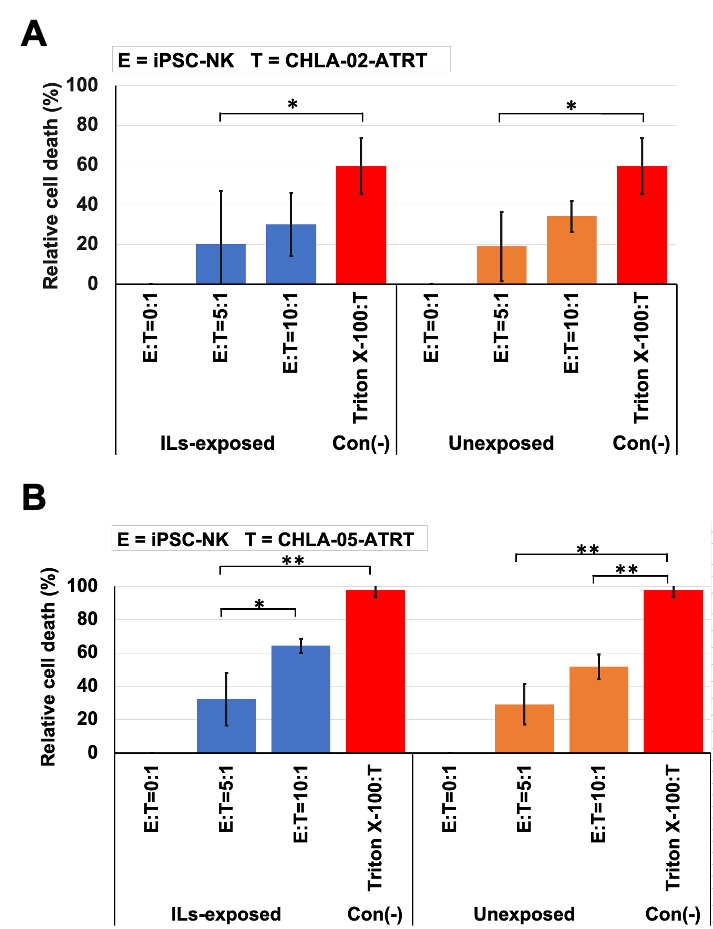


**Supplementary Figure S4: Calcein-AM-based cytotoxicity assay to determine the specific cell death percentage of ATRT cells. (A)** CD56^bright^ CD16^bright^ iPSC-NK cells as effectors. **(B)** NK92mi cells as effectors. Statistical significance is denoted by * and ** for *p*-values less than 0.05 and 0.01, respectively. The symbol "∆" indicates a significant difference between ILs-exposed and unexposed groups at the same E:T ratio. ILs: IL2, IL12, IL15, IL18, and IL21.


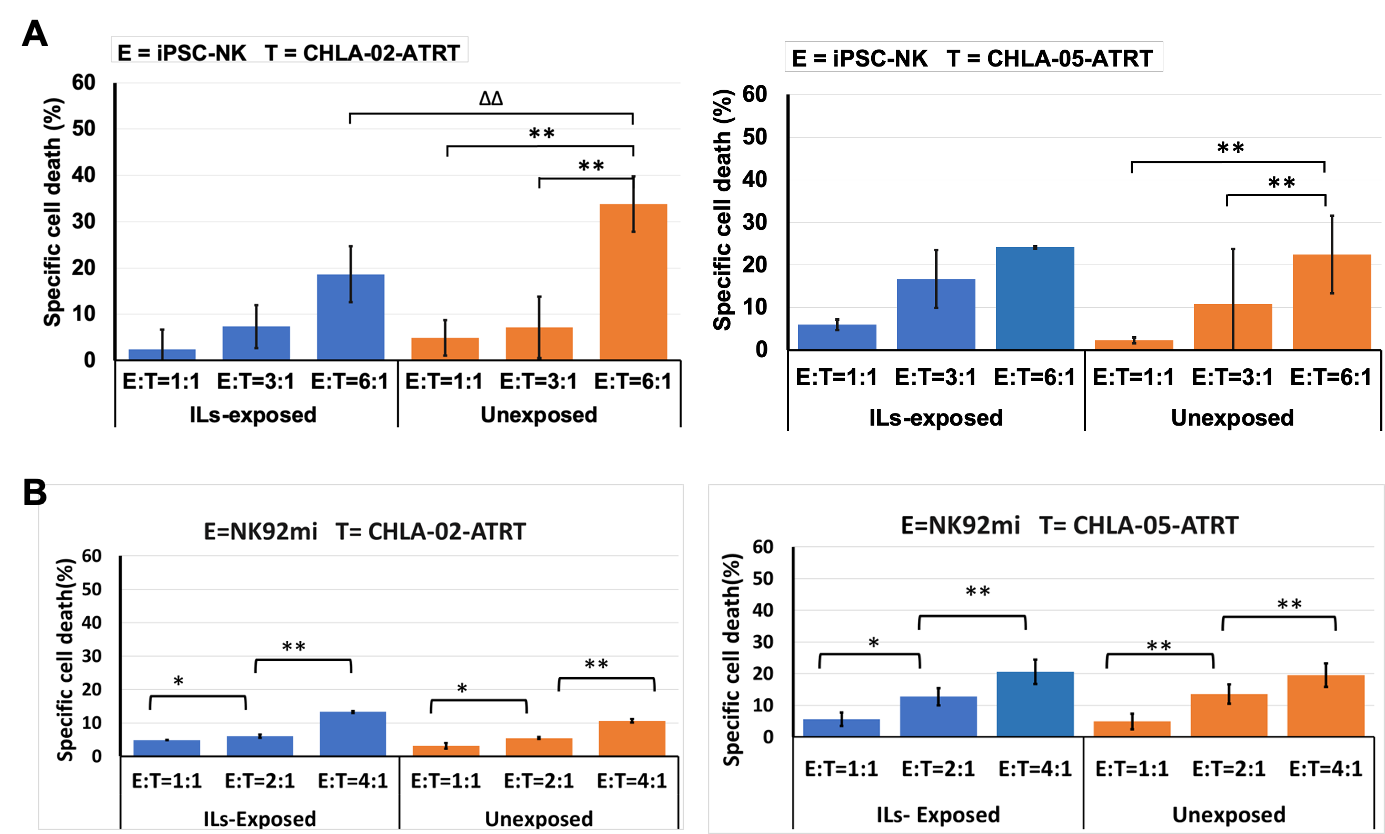


**Supplementary Figure S5. The examination of subpopulations existing at the CD56^bright^ CD16^bright^ stage through immunocytochemistry. (A)** Lymphoid progenitor markers, CD7, ZBTB-16, CD5, and GATA3. Natural killer T cell or other lymphoid lineage markers, CD56 and CD3D. **(B)** Natural killer cell marker, CD56, CD16, TRAIL, NKp30, NKG2D, and CD158a. ZBTB-16, Zinc finger and BTB domain containing 16; GATA3, Trans-acting T-cell-specific transcription factor GATA3; CD3D, T-cell surface glycoprotein CD3 delta chain; TRAIL, TNF-related apoptosis-inducing ligand; NKp30, Natural killer cell p30-related protein; NKG2D, NKG2-D type II integral membrane protein. **Scale bar, 100 µm.**


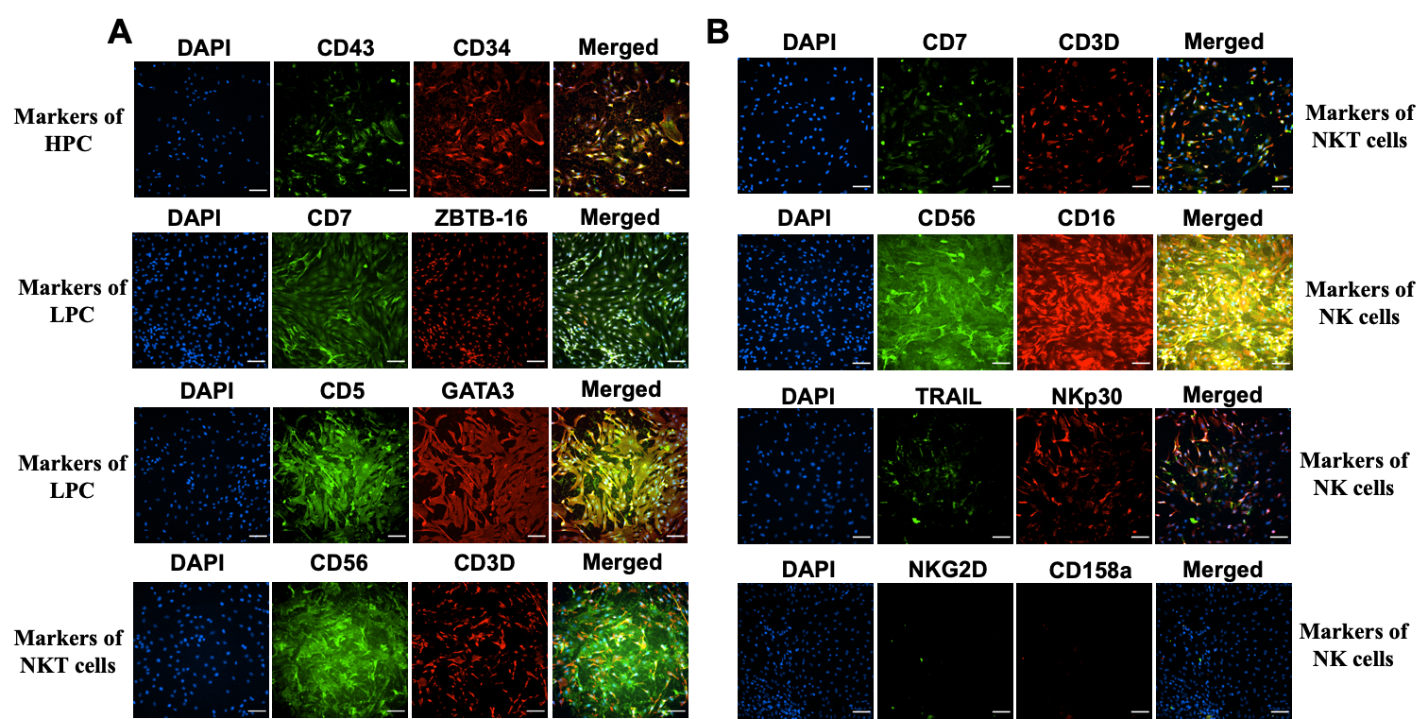


**Supplementary Figure S6: Gating events of CD56^-ve^ CD16^bright^ cells and an activation marker in CD56^bright^ CD16^bright^ cells.** The gating events for Figure 8C, further maturation of iPSC-NK cells into CD56^-ve^ CD16^bright^ phenotype. Markers were detected after exposure to IL-15, ILs (IL-2, IL-12, IL-15, IL-18, and IL-21), or CHLA-02-ATRT.


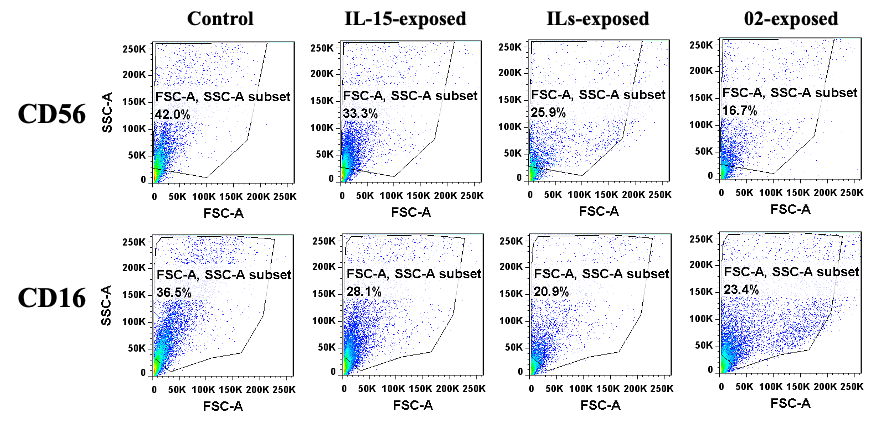


**Supplementary Table S1.** **A brief description of NK cell phenotype, activation, and cytotoxicity markers.** CD: Cluster of differentiation, ZBTB: Zinc Finger and BTB Domain; PLZF: Promyelocytic leukemia zinc finger protein; KIR2DL1: Killer-cell immunoglobulin-like receptors; NKG2D: Natural Killer Group 2D; LAMP1: Lysosome-associated membrane glycoprotein 1; IFN-γ: Interferon γ; TNF: Tumor Necrosis Factor.

| **Markers** | **Specificity** |
| --- | --- |
| CD56 | NK cell-specific marker |
| CD34, CD43 | Hematopoietic progenitor markers |
| CD45 | Common hematopoietic cell marker |
| ZBTB16/PLZF | Innate cell immunity |
| CD3 | T cell co-receptor |
| CD16 | Expressed in large granular lymphocytes |
| CD94 | CD94/NKG2 inhibitory receptors in developing NK |
| KIR2DL1/CD158a | Killer cell inhibitory receptor expressed in subset of NK |
| NKG2D | Activating receptors of NK cell |
| Activating receptors of NK cell |  |
| NKP30 |  |
| LAMP1/CD107a | LAMP1/CD107a |
| CD107a | Stimulated NK cells |
| IFN-𝛾 |  |
| TNF-⍺ |  |

**Supplementary Table S2. List of antibodies.**

| **Antibodies** | **Origin/Isotype** | **Supplier/Cat #** | **Dilution for ICC and flow cytometry** |
| --- | --- | --- | --- |
| **CD34** | Rabbit IgG | Sino, 10103-R009 | 1:700 |
| **CD43** | Rabbit IgG | Sino, 101324-T36 | 1:200 |
| **CD43** | Mouse IgG | R&D system, MAB20381 | 1:100 |
| **CD7** | Mouse IgG | Sino, 11028-MM14 | 1:25-1:100 |
| **ZBTB16** | Rabbit IgG | Sino, 200261-T32 | 1:200 |
| **CD56** | Mouse IgG | STEMCELL, 60021 | 1:20 |
| **CD16** | Rabbit IgG | Sino, 10389-R221 | 1:20 |
| **GATA3** | Rabbit IgG | Invitrogen, PIPA581181 | 1:50 |
| **CD5** | Mouse IgG | R&D system, MAB16361 | 1:100 |
| **CD25** | FITC-Rabbit IgG | Sino, 10165-R216-F | 1:20 |
| **CD94** | Mouse IgG | BioLegend, 305502 | 1:100 |
| **CD3E+CD3G** | Rabbit IgG | Sino, CT026-R301 | 1:20 |
| **Perforin** | FITC-Mouse IgG | BioLegend, 308104 | 1:20 |
| **NKG2D** | Mouse IgG | Sino, 10575-MM02 | 1:100 |
| **KIR2DL1** | Rabbit IgG | Sino, 13145-R124 | 1:100 |
| **CD107a** | Rabbit IgG | Sino, 11215-R107 | 1:100 |
| **SH2D1A** | Rabbit IgG | ABclonal, A1143 | 1:50 |
| **NKp46** | Rabbit IgG | BioLegend, 331902 | 1:200 |
| **TNF⍺** | Rabbit IgG | Sino, 10602-R101 | 1:50 |
| **IL-6** | Mouse IgG | Sino, 10395-MM10 | 1:100 |
| **IFN𝛾** | Mouse IgG | Sino, 11725-M005 | 1:250 |
| **NKp30** | Rabbit IgG | Sino 10480-R012 | 1:200 |
| **Alexa Fluor^TM^ 488** | Goat Anti-Mouse IgG, IgM, IgA (H+L) | Invitrogen, A-10667 | 1:200 |
| **Alexa Fluor^TM^ 594** | Goat Anti-rabbit IgG (H+L) | Invitrogen, A-11012 | 1:200 |
| **Alexa Fluor^TM^**  **568** | Goat anti-Mouse IgG (H+L) | Invitrogen, A-11004 | 1:200 |

**Supplementary Table S3. Sequences of forward and reverse primers for RT-PCR.**

| **Gene** | **Orientation** | **Sequences (5’ to 3’)** |
| --- | --- | --- |
| **CD16** | F | CAAATCCTTCATCATGTCAGTTCC |
|  | R | GAACCAAGAAATGTTGCGCTTA |
| **CD56** | F | GCAGATGGGAGAGGATGGAAA |
|  | R | TCTGGTTTCCACTCGGAGGAG |
| **NKG2D** | F | TCAAGATCTTCCCTCTCTGAGCA |
|  | R | CGAATCCACCCCATCAAATAC |
| **NKp46** | F | ACTAGGCCGGCAGAATCTGAG |
|  | R | CGAGAGGGTGGGTGTGTCATA |
| **CD107a** | F | TCACACGTAGGACGCATGAAG |
|  | R | GAAGCGCTCCAGACACTCATC |
